# Transcription Profiling Reveals Cooperative Metabolic Interactions in a Microbial Cheese-Ripening Community Composed of *Debaryomyces hansenii*, *Brevibacterium aurantiacum*, and *Hafnia alvei*

**DOI:** 10.3389/fmicb.2019.01901

**Published:** 2019-08-16

**Authors:** Nguyen-Phuong Pham, Sophie Landaud, Pascale Lieben, Pascal Bonnarme, Christophe Monnet

**Affiliations:** UMR GMPA, AgroParisTech, INRA, Université Paris-Saclay, Thiverval-Grignon, France

**Keywords:** *Brevibacterium aurantiacum*, *Hafnia alvei*, *Debaryomyces hansenii*, cheese, microbial communities, biotic interactions, siderophore, galactonate

## Abstract

Ripening cultures containing fungi and bacteria are widely used in smear-ripened cheese production processes, but little is known about the biotic interactions of typical ripening microorganisms at the surface of cheese. We developed a lab-scale mini-cheese model to investigate the biotic interactions of a synthetic community that was composed of *Debaryomyces hansenii*, *Brevibacterium aurantiacum*, and *Hafnia alvei*, three species that are commonly used for smear-ripened cheese production. Transcriptomic analyses of cheese samples produced with different combinations of these three species revealed potential mechanisms of biotic interactions concerning iron acquisition, proteolysis, lipolysis, sulfur metabolism, and D-galactonate catabolism. A strong mutualistic interaction was observed between *H. alvei* and *B. aurantiacum*. We propose an explanation of this positive interaction in which *B. aurantiacum* would benefit from siderophore production by *H. alvei*, and the latter would be stimulated by the energy compounds liberated from caseins and triglycerides through the action of the proteases and lipases secreted by *B. aurantiacum*. In the future, it would be interesting to take the iron acquisition systems of cheese-associated strains into account for the purpose of improving the selection of the ripening culture components and their association in mixed cultures.

## Introduction

Microbial communities from rinds of smear-ripened cheeses contain various types of bacteria and fungi that contribute to the development of cheese flavor, texture, and appearance (Beresford et al., 2001; Larpin-Laborde et al., 2011; Cogan et al., 2014; Irlinger et al., 2015; Fox et al., 2017). Traditionally, the production of smear-ripened cheeses involves a common step of “old-young smearing” in which a smear fluid taken from mature (“old”) cheeses is used to inoculate the surface of freshly made (“young”) cheeses. However, selected ripening cultures are often used today in the cheese industry to standardize manufacturing processes, to provide specific sensory properties and to outcompete pathogens or spoilage microorganisms (Bockelmann, 2010). These ripening cultures typically contain three to six strains. They are composed of fungi such as *Debaryomyces hansenii* and *Geotrichum candidum* whose main function is to raise the pH of the cheese curd, and of acid-sensitive ripening bacteria that begin to grow when the pH rises to above approximately 5.5 to 6.0, and whose main function is to produce aroma compounds and pigments. For now, the design of ripening cultures is rather empirical and it would be interesting to better take the biotic interactions between the typical strains that grow at the surface of cheese into account. Except for the positive impact of the pH increase initiated by fungi on the growth of acid-sensitive bacteria, little is known about the main biotic interactions that occur at the cheese surface.

Over the past few years, several studies demonstrated the feasibility and benefits of microbial transcriptomic analyses of cheeses (Lessard et al., 2014; Dugat-Bony et al., 2015; De Filippis et al., 2016; Monnet et al., 2016; Duru et al., 2018). Recent technical advances and the reduced cost of high-throughput sequencing technologies (HTS) now allow researchers to accurately capture the transcriptomes of two or more species present in the same sample. This relatively new approach, known as dual RNA-seq, offers a more complete prospect for dissecting interspecies interactions (Wolf et al., 2018).

The aim of this study was to investigate, by a combination of microbial, biochemical, and transcriptomic analyses, the biotic interactions in lab-scale mini-cheeses produced with a ripening culture composed of three microorganisms: *D. hansenii*, whose function is to increase the pH, *Brevibacterium aurantiacum* and *Hafnia alvei*. The latter two species are able to produce volatile sulfur compounds (VSCs) and are sometimes associated together in commercial cultures used for cheese manufacturing processes in which a high production level of aroma compounds is desired. *B. aurantiacum* is also frequently used in ripening cultures for its capacity to produce orange pigments in cheeses.

## Materials and Methods

### Strains and Growth Conditions

*Debaryomyces hansenii* 304, *B. aurantiacum* 8(6), and *H. alvei* GB001 were from the GMPA culture collection (INRA, Thiverval-Grignon, France). All these strains were originally isolated from cheeses. The yeast *D. hansenii* was grown in potato dextrose broth (BD Difco^TM^; Becton, Dickinson and Company, Sparks, MD, United States), and the bacteria were grown in brain heart infusion broth (Biokar Diagnostics, Beauvais, France). Two successive cultures were performed before inoculation of the cheese curds. In the first culture, the strains were grown under aerobic conditions (rotary shaker at 250 rpm) at 25°C in 50-ml conical flasks containing 10 ml of growth medium. After incubation for 48 h, a 250-ml conical flask containing 50 ml of the corresponding medium was inoculated with 1 ml of the previous culture and incubated for 24 h under the same conditions.

### Lab Scale Mini-Cheese Production

The curd used for the model cheese production was a non-brined curd provided by an industrial cheesemaker. It was manufactured from pasteurized milk using a mesophilic starter culture composed of *Lactococcus* strains (CHOOZIT^TM^ MA 19, DuPont Danisco, Sassenage, France), and according to the typical Raclette-type manufacturing process. Blocks of approximately 500 g were wrapped in plastic bags and stored at −20°C. The curd blocks were thawed for 24 h at + 4°C and grated with an electric grater. A saline solution (NaCl 70.1 g/l) was added to the curd at a final concentration equivalent to 1.7 g NaCl/100 g, and the mixture was then homogenized for 5 min at 24,000 rpm with a mechanical blender (Ultra-Turrax model T25; IKA Labortechnik, Staufen, Germany). The salted curd was autoclaved for 15 min at 108°C, and the subsequent stages of model cheese production were carried out under aseptic conditions. The sterilized curd was mixed and subsequently cooled to 20°C. The cultures of the yeast and bacteria were centrifuged at 4,500 × *g* for 10 min at 4°C, cells were washed and re-suspended in physiological water (NaCl 9 g/l) and added to the curd at the final concentration of 10^4^ (yeast) or 10^6^ (bacteria) CFU/g. Inoculated curd was then transferred onto a circular plastic grid (diameter: 2.7 cm), which rested on a disposable cap (Kim-Kap^TM^, DWK Life Sciences, Rockwood, TN, United States) in a disposable 40-ml sample container (Gosselin^TM^, Dominique Dutscher, Brumath, France). Average cheese size was approximately 2.7 cm in diameter and 1.0 cm thick, with an average weight of 6.2 g. These mini-cheeses are mainly representative of the cheese surface due to the low thickness of the loaf. The container cap was unscrewed (three-quarters of a turn) to allow air exchange, and the mini-cheeses were placed in an incubator at 15°C.

In order to investigate the biotic interactions in a ripening culture composed of *D. hansenii*, *B. aurantiacum*, and *H. alvei*, we compared different mini-cheeses that were produced with the complete community, with *D. hansenii* alone, with *D. hansenii* and *H. alvei*, and with *D. hansenii* and *B. aurantiacum*, ripened at 15°C for 21 or 28 days. In total, eight different biological conditions were investigated (Table 1). The combinations in which the yeast is omitted were not considered here since no growth of the bacteria occurred due to the acidity of the cheese curd (pH = 5.1). Four cheese replicates (*N* = 4) were performed for each biological condition.

**TABLE 1:** Species combinations investigated in this study.

**Condition code**	**Culture composition**			**Ripening time at 15°C**
		
	***D. hansenii***	***H. alvei***	***B. aurantiacum***	
D21_DH	+	−	−	21 days
D21_DH_HA	+	+	−	
D21_DH_BA	+	−	+	
D21_DH_HA_BA	+	+	+	
D28_DH	+	−	−	28 days
D28_DH_HA	+	+	−	
D28_DH_BA	+	−	+	
D28_DH_HA_BA	+	+	+	

### Microbial Analyses

Each mini-cheese was mixed with a spatula, and 0.5 g of sample was then mixed with 9.5 ml of physiological water (9 g/l NaCl). After homogenization with an Ultra-Turrax blender for 1 min at 20,500 rpm, 10-fold serial dilutions were prepared in physiological water and plated in duplicate on agar plates. *D. hansenii* colonies were counted on yeast extract-glucose-chloramphenicol agar (Biokar Diagnostics) after 3 days of incubation at 25°C. Colonies of bacteria were counted on brain heart infusion agar (Biokar Diagnostics) supplemented with 50 mg/l amphotericin (Sigma-Aldrich, St. Louis, MO, United States), which inhibits the growth of fungi, after 3 days of incubation at 25°C. *B. aurantiacum* and *H. alvei* could be differentially counted on this medium because of their distinct color of colonies. Microbial growth was calculated as the arithmetic mean of all biological replicates (*N* = 4), and significant differences between conditions were evaluated by Student’s *t*-test. Absence of contamination was checked by examination of the morphotypes of the colonies grown on agar plates.

### Measurement of pH and Evaluation of Proteolytic and Lipolytic Activities

The pH values were measured on the mini-cheeses that were mixed with a spatula. Proteolytic activity was determined on calcium caseinate agar modified according to Frazier and Rupp (Merck, Darmstadt, Germany) supplemented with 1% (w/v) skim milk powder (BD Difco^TM^). Lipolytic activity was determined on tributyrin agar (Sigma-Aldrich) supplemented with 1% (w/v) tributyrin (Sigma-Aldrich). Ten microliters of cell suspensions at 10^6^ CFU/ml in physiological water (NaCl 9 g/l) were spot-inoculated onto the plates, which were then incubated at 15 or 25°C for 21 days. Proteolytic and lipolytic activities were evaluated by the formation of a clear zone around the spots.

### Metabolite Extraction and Analysis by UHPLC-MS and HPLC-UV

All cheeses were frozen at −20°C until extraction. The metabolite extraction procedure was adapted from the method previously described by Le Boucher et al. (2013). Briefly, cheese samples of approximately 2 g were 10-fold (w/w) diluted in deionized water (Milli-Q Reagent Grade Water System, Millipore Corporation, Billerica, MA, United States). The mixture was then homogenized with an Ultra-Turrax blender for 1 min at 20,500 rpm, and the homogenate was centrifuged at 10,000 × *g* for 10 min at 4°C. The supernatant was recovered and centrifuged for 30 min at 8000 × *g* and 4°C on centrifugal filter units with a molecular cut-off of 10 kDa (Vivaspin 20, Sartorius, Palaiseau, France).

The filtrate was then diluted in 1% formic acid for analyses by UHPLC-MS (UHPLC Ultimate 3000 and HR-MS-Q EXACTIVE, Thermo Fisher Scientific, France). UHPLC conditions were as follows: metabolites were separated on a Hypersil GOLD phenyl column (length = 15 mm, internal diameter = 2.1 mm, particle size = 3 μm; Thermo Fisher Scientific, France). The pressure at the beginning of the gradient was 120 bars and the column temperature was 25°C. The flow was 0.25 ml/min and the solvents were D: acetonitrile (HPLC quality) and B: ultra-pure water + nonafluoropentanoic acid (3 mM). The elution gradient was as follows: 4 min at 98% B + 2% D, then 98% to 2% B in D for 6 min and, finally, 2% B and 98% D for 3 min. The injection volume was 5 μl and the injector temperature was 7°C. The duration of one analysis was 14 min. Mass spectrometric detection was performed with a quadrupole-orbitrap with an electrospray source operated in the positive ionization mode. Full scans were acquired with a scan range of 3.7 scan/sec and a mass range from 50 to 700 u.m.a. (unified atomic mass unit) with a resolution of 70,000. Data were identified and quantified using TraceFinder software (Thermo Fisher Scientific) according to the calibration solution.

Lactose, citric acid, galactose, lactic acid, glycerol, acetic acid, and ethanol were determined by HPLC (Waters S.A.S., Saint-Quentin, France). Separation was performed on a Bio-Rad Aminex HPX-87H column (300 mm × 7.8 mm; Bio-Rad, Richmond, CA, United States), equipped with a cation H1 Micro-Guard column (30 mm × 4.6 mm; Bio-Rad) at an eluent flow rate of 0.6 ml/min (e2695 pump; Waters S.A.S.) and a temperature of 35°C. 5 mM H_2_SO_4_ was used as an eluent. Quantification was performed using a 410 refractive index (RI) detector and a 2489 UV/Visible detector (Waters S.A.S.) at 210 nm, with external standards (Sigma) of known amounts of commercially pure substances prepared fresh in filtered, deionized water. Results were analyzed by EmPOWER software (Waters S.A.S.).

### Extraction of RNA From Cheese Samples, rRNA Depletion and RNA Sequencing

RNA was extracted from 500-mg mini-cheese samples without prior separation of microbial cells, as previously described (Monnet et al., 2012), except that UptiZol reagent (Interchim, Montluçon, France) was used instead of TRIzol, the Phase Lock Gel tubes were replaced by MaXtract High Density tubes (Qiagen, Germantown, MD, United States), and bead beating was performed on a Precellys Evolution bead beater (Bertin, Montigny-le-Bretonneux, France) using two 20-s mixing sequences at a speed of 10,000 rpm. In addition, in order to increase the concentration of RNA, the content of two RNA extraction tubes from the same mini-cheese sample was pooled after the bead-beating step. Purified RNA was quantified with Qubit RNA assay kits on the Qubit 3.0 fluorometer (Life Technologies, Carlsbad, CA, United States), and RNA quality was analyzed with an Agilent model 2100 Bioanalyzer (Agilent, Palo Alto, CA, United States) using RNA 6000 NANO chips, according to the manufacturer’s instructions. The concentration of RNA was adjusted to 100 ng/μl with RNase-free water, and DNase treatment was performed using TURBO^TM^ DNase (Invitrogen, Carlsbad, CA, United States) according to the manufacturer’s instructions. A second DNase treatment was performed on 1000 ng of total RNA (Baseline Zero DNase, Epicentre), and rRNA was then depleted using the Epicentre Ribo-Zero^TM^ Magnetic Gold Kit (Tebu-bio, Le Perray-en-Yvelines, France) for bacteria (reference MRZB12424) and for yeasts (reference MRZY1324), as previously described (Monnet et al., 2016). Directional RNA-seq libraries were constructed using the ScriptSeq V2 RNA-seq library preparation kit (Illumina), according to the manufacturer’s recommendations (11 PCR cycles were performed). Libraries were pooled in equimolar proportions and sequenced (Single Read 75 pb) on an Illumina NextSeq500 instrument, using a NextSeq 500 High Output 75 cycles kit. Demultiplexing was done with bcl2fastq2 conversion software (V2.2.18.12), and adapters were trimmed with Cutadapt 1.15 (Martin, 2011). Reads with a size of less than 10 bp after trimming were discarded.

### Mapping Against Reference Genomes

Sequencing reads were mapped against the appropriate reference database using Bowtie short read aligner version 1.2.1.1 (Langmead et al., 2009) with the following parameters: -a -m 1 –best –strata -v 2 -t -S. A maximum of two mismatches was allowed for each sequencing read. The reference databases were composed of the coding DNA sequences (CDSs) of the strains that were inoculated in the mini-cheeses, except for *D. hansenii*, for which mapping was done on the genome of *D. hansenii* CBS767. The NCBI BioProject accession numbers of the strains used for the mapping are: PRJEB19868 [*B. aurantiacum* 8(6)], PRJEB6257 (*H. alvei* GB001), and PRJNA13832 (*D. hansenii* CBS767). The numbers of reads that mapped onto the reference genomes were counted using HTSeq-count version 0.10.0 (Anders et al., 2015) with the following parameters: -s yes -t CDS -i locus_tag -m union. Only reads that mapped to unique sequences were further analyzed.

### RNA-seq Data Analyses

Sequencing reads that mapped to the CDSs databases were retrieved from the raw dataset. Data were filtered to remove genes displaying an average of <10 reads per sample across the dataset. Normalization was performed against each species: for each sample, the read numbers were divided by the sum of the reads that mapped to the CDSs of the corresponding species and multiplied by a factor 1,000,000 (bacteria) or 10,000,000 (yeast). Functional classification of the transcriptomic dataset was performed using the Kyoto Encyclopedia of Genes and Genomes (KEGG) annotations (Kanehisa et al., 2012). Differential expression analysis was conducted using the DESeq2 package (Love et al., 2014) implemented in Galaxy^1^. Raw *p*-values were adjusted for multiple testing using the Benjamini-Hochberg procedure (Benjamini and Hochberg, 1995), which assesses the False Discovery Rate. Gene transcripts with an adjusted *p* < 0.05 were considered to be differentially expressed between two conditions.

## Results

### Microbial Growth in the Mini-Cheeses

**FIGURE 1:**
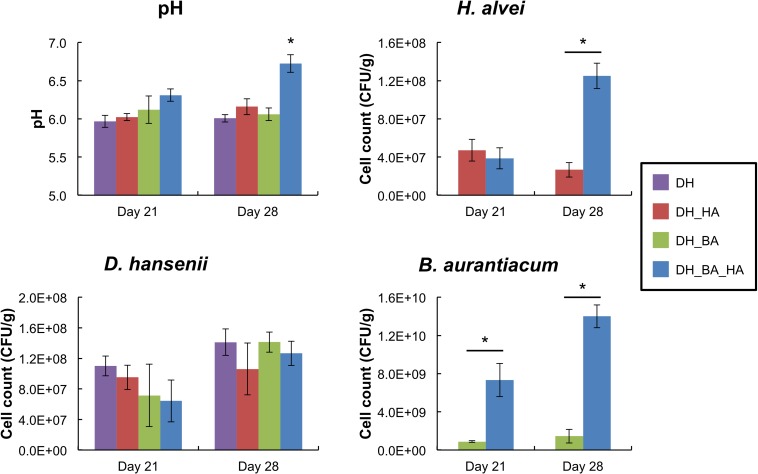
pH values and growth of *Debaryomyces hansenii*, *Hafnia alvei*, and *Brevibacterium aurantiacum* in the mini-cheeses at day 21 and day 28. Bars are colored according to the biological conditions; DH, HA, and BA correspond to the presence of *D. hansenii*, *H. alvei*, and *B. aurantiacum*, respectively. The error bars represent the standard deviations (four cheese replicates), and the asterisks indicate a significant difference compared to the other biological conditions at the same sampling time (*p* < 0.05, *t*-test).

There was no significant impact of the presence of *B. aurantiacum* and/or *H. alvei* on the cell counts of *D. hansenii* at day 21 and day 28 (Figure 1). However, the presence of *H. alvei* stimulated the growth of *B. aurantiacum* (about eightfold at day 21, and 10-fold at day 28), and the latter stimulated *H. alvei* at day 28 (about fivefold). There was thus a mutualistic interaction between the two bacteria. At day 28, the pH values of the mini-cheeses were also higher when the two bacteria were present together (pH = 6.73 vs. 6.01 to 6.16 in the other conditions; *p* < 0.05).

### Overview of the Transcriptomic Data

RNA-seq analyses of the mini-cheeses were performed in order to gain insight into the potential molecular mechanisms underlying the biotic interactions between the investigated microorganisms. The mean numbers of sequencing reads generated in the four mini-cheese replicates of the eight biological conditions were between 76 and 89 million, and between 58.8 and 64.6% of the sequencing reads mapped to unique sequences of the CDS databases (Table 2). *D. hansenii* accounted for most of the mapped reads (Supplementary Figure S1). The number of sequencing reads mapped to the *B. aurantiacum* genome was higher in the presence of *H. alvei* (an approximate sixfold increase at day 21 and a 15-fold increase at day 28), which was probably due to the growth stimulation of *B. aurantiacum* by *H. alvei*. There was also a higher proportion of detected CDSs (the detection cut-off was set to a mean number of ten reads per CDS in the cheese replicates) in *B. aurantiacum* when it was co-cultivated with *H. alvei* (Supplementary Table S1).

**TABLE 2:** Statistics of sequencing read mapping to the CDSs databases.

**Biological condition^a^**	**Total reads^b^**	**Mapped reads (unique sequences)**	**% Mapped reads (unique sequences)**	**Mapped to *D. hansenii* CDSs**	**Mapped to *H. alvei* CDSs**	**Mapped to *B. aurantiacum* CDSs**
D21_DH	79,905,467	50,257,046	62.9	50,257,046	–	–
D28_DH	83,733,859	53,757,920	64.2	53,757,920	–	–
D21_DH_HA	80,852,459	50,375,856	62.3	47,039,407	3,336,449	–
D28_DH_HA	81,811,905	52,847,087	64.6	48,235,272	4,611,815	–
D21_DH_BA	88,754,767	55,922,116	63.0	55,155,168	–	766,948
D28_DH_BA	77,531,552	49,641,338	64.0	49,193,119	–	448,219
D21_DH_HA_BA	82,490,590	50,891,718	61.7	44,784,521	1,918,278	4,188,919
D28_DH_HA_BA	76,757,851	45,158,865	58.8	34,728,532	3,565,966	6,864,367

*^*a*^Biological conditions are coded according to the following rules: D21 and D28 correspond to the sampling time (day 21 and day 28, respectively); DH, HA, and BA correspond to the presence of *D. hansenii*, *H. alvei*, and *B. aurantiacum*, respectively. ^*b*^Mean of the reads from the four cheese replicates.*

The transcriptomes of the individual species were normalized against the corresponding species (the read numbers for each CDS were divided by the sum of the reads that mapped to CDSs of the species) and compared in the different biological conditions. For each of the three species, the global distribution of the KEGG categories within the transcriptomes was quite similar in the different biological conditions (Supplementary Figure S2). However, for *B. aurantiacum*, co-cultivation with *H. alvei* decreased the proportion of sequencing reads from the KEGG category “Membrane transport.”

### Differential Expression Analysis of the Transcriptomes

Differential expression analyses of the transcriptomes were performed for each of the three species in the different biological conditions (Supplementary Table S2). The presence of *H. alvei* impacted the transcriptome of *B. aurantiacum*, and vice versa. There was a much higher impact of the combination of *H. alvei* and *B. aurantiacum* on the transcriptome of *D. hansenii* than when only one of these bacteria was inoculated in the mini-cheeses. The metatranscriptomic data provided a large amount of information regarding the biotic interactions between the investigated species during their growth in the mini-cheeses. The most interesting findings are described below.

#### Catabolism of D-Galactonate, Lactate, Acetate, Ethanol, and Citrate

**FIGURE 2:**
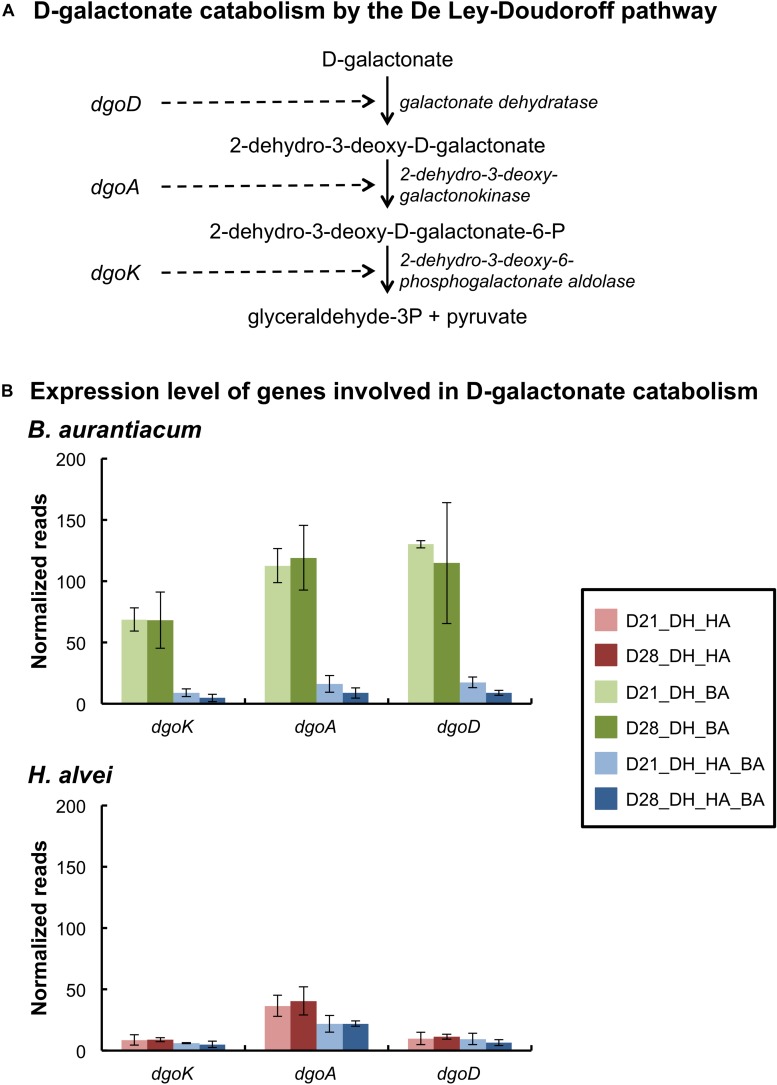
Expression of the D-galactonate catabolism pathway of the bacteria in the mini-cheeses. **(A)** Scheme of D-galactonate catabolism by the De Ley-Doudoroff pathway. **(B)** Expression levels of the genes involved in D-galactonate catabolism in *B. aurantiacum* and *H. alvei*. Normalization was performed against each species: the read numbers were divided by the sum of the reads that mapped to the CDSs of the corresponding species and multiplied by a factor of 1,000,000. Bars are colored according to the biological conditions; D21 and D28 correspond to the sampling time (day 21 and day 28, respectively); DH, HA, and BA correspond to the presence of *D. hansenii*, *H. alvei*, and *B. aurantiacum*, respectively. The error bars represent the standard deviations (four cheese replicates).

*Brevibacterium aurantiacum* expressed the genes encoding the enzymes of the D-galactonate catabolism pathway, and these genes were strongly repressed in the presence of *H. alvei* (Figure 2 and Supplementary Table S4). The latter species also expressed the enzymes of this pathway (Figure 2 and Supplementary Table S5). D-galactonate was detected in the cheese curd before inoculation at a concentration of 10.3 ± 0.3 μmol/kg by UHPLC-MS analysis, and it increased to 56.0 ± 3.1 μmol/kg in the mini-cheeses inoculated with *D. hansenii* at day 28 (Supplementary Table S3). These results suggest that some D-galactonate is produced in the mini-cheeses by *D. hansenii* and that this compound is probably catabolized by *B. aurantiacum* and *H. alvei*. *H. alvei* increased the expression of the genes encoding the three-subunit quinone/cytochrome-dependent L-iLDH complex LldEFG in *B. aurantiacum* (Supplementary Table S4). This enzyme catalyzes the oxidation of lactate, which is an energy source for many ripening bacteria. We also observed that the *H. alvei citTGXFEDC* gene cluster, which encodes the enzymes involved in citrate catabolism, was repressed in the presence of *B. aurantiacum* (Supplementary Table S5).

#### Nitrogen Metabolism

**FIGURE 3:**
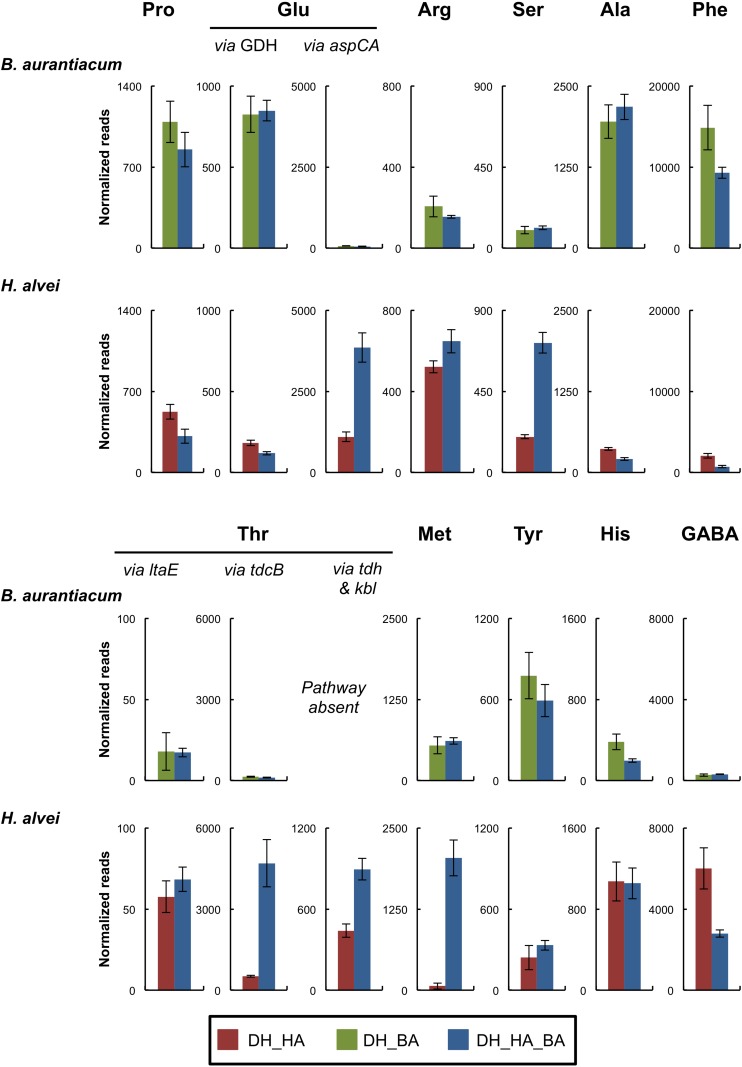
Expression of amino acid catabolism pathways of the bacteria in the mini-cheeses at day 28. The expression level of each pathway is represented as the sum of the sequencing reads (normalized against each species) that mapped to the genes of the corresponding pathway. Bars are colored according to the biological conditions; DH, HA, and BA correspond to the presence of *D. hansenii*, *H. alvei*, and *B. aurantiacum*, respectively. The error bars represent the standard deviations (four cheese replicates). Pro, proline; Glu, glutamate; Arg, arginine; Ser, serine; Ala, alanine; Phe, phenylalanine; Thr, threonine; Met, methionine; Tyr, tyrosine; His, histidine; GABA, gamma-aminobutyrate; GDH, glutamate dehydrogenase; *aspC*, aspartate aminotransferase; *aspA*, aspartate ammonia-lyase; *ltaE*, L-threonine aldolase; *tdcB*, threonine ammonia-lyase; *tdh*, L-threonine 3-dehydrogenase; *kbl*, 2-amino-3-ketobutyrate coenzyme A ligase.

Contrary to *D. hansenii* and *H. alvei*, *B. aurantiacum* had a proteolytic activity on calcium caseinate agar (Table 3). *H. alvei* had no major impact on the expression of putative excreted proteases/peptidases in *B. aurantiacum* (Supplementary Table S6). The presence of *H. alvei* decreased the expression of the phenylalanine catabolic pathway in *B. aurantiacum*, and vice versa (Figure 3 and Supplementary Tables S7, S8). Interestingly, co-cultivation of *H. alvei* with *B. aurantiacum* considerably increased its expression of two genes encoding methionine gamma-lyase, an enzyme involved in methionine catabolism (Supplementary Table S8). This suggests that there is a higher methionine availability for *H. alvei* when *B. aurantiacum* is present. This is in agreement with the metabolomic analyses, which revealed a higher concentration of methionine at day 28 in the mini-cheeses inoculated with *D. hansenii*, *H. alvei*, and *B. aurantiacum*, in comparison to the mini-cheeses inoculated with only *D. hansenii* and *H. alvei* (Supplementary Table S3). In addition, the presence of *B. aurantiacum* increased the expression of the threonine degradation pathways via threonine dehydrogenase and via threonine ammonia-lyase, of the serine degradation pathway via L-serine ammonia-lyase, and of the glutamate degradation pathway via aspartate aminotransferase in *H. alvei*. The expression of the *H. alvei gabT* and *gabD* genes, which are involved in gamma-aminobutyrate (GABA) degradation, decreased in the presence of *B. aurantiacum* (Supplementary Table S8). In *Escherichia coli*, these two genes are induced by nitrogen limitation (Schneider et al., 2002). It can therefore be hypothesized that the presence of *B. aurantiacum* increased the availability of nitrogen sources for *H. alvei*.

**TABLE 3:** Proteolytic and lipolytic activities of the strains used in this study.

**Species**	**Strain**	**Proteolytic activity^a^**		**Lipolytic activity^b^**	
			
		**15°C**	**25°C**	**15°C**	**25°C**
**Bacteria**					
*B. aurantiacum*	8 (6)	+ +	+	++	+ +
*H. alvei*	GB001	0	0	0	+
**Yeast**					
*D. hansenii*	304	0	0	+ +	++

*^*a*^Determined on calcium caseinate agar. ^*b*^Determined on tributyrin agar. All tests were carried out in triplicate. 0, no halo was observed; +, radius of proteolytic/lipolytic zone < 2 mm; + ⁣ +, 2–4 mm; the radius was measured from the edge of the spot to the edge of the halo.*

The expression of several *D. hansenii* genes involved in nitrogen metabolism was impacted by the presence of *H. alvei* and *B. aurantiacum* in the mini-cheeses (Supplementary Table S9). Interestingly, when *H. alvei* and *B. aurantiacum* were simultaneously inoculated into the mini-cheeses, there was a higher expression level of the pathways for glutamate degradation to alpha-ketoglutarate, for glutamate degradation to GABA, and for GABA degradation to succinate (Supplementary Figure S3). This suggests that the combination of *H. alvei* and *B. aurantiacum* increased the availability of glutamate for *D. hansenii*, which was in agreement with the metabolomic analyses, revealing a much higher concentration of glutamate in the corresponding samples (Supplementary Table S3). Furthermore, in comparison to the mini-cheeses inoculated with only *D. hansenii*, the addition of both *H. alvei* and *B. aurantiacum* to the mini-cheeses decreased the expression of the *D. hansenii* genes involved in the utilization of two other nitrogen sources: ammonium and urea. In *Saccharomyces cerevisiae*, urea is considered as a poor nitrogen source, and its uptake and catabolism is repressed via the nitrogen catabolism repression (NCR) process in the presence of rich nitrogen sources (Hofman-Bang, 1999). Higher nitrogen availability was confirmed here by the metabolomic analyses, which showed that the mini-cheeses simultaneously inoculated with *H. alvei* and *B. aurantiacum* had the highest total concentration of free amino acids among all the investigated conditions (Supplementary Figure S4 and Supplementary Table S3).

#### Lipid Catabolism

*Brevibacterium aurantiacum* and *D. hansenii* had a lipolytic activity on tributyrin agar at 15°C, but no activity was detected for *H. alvei* (Table 3). This was in accordance with genomic analyses, which revealed that only the *H. alvei* genome did not encode a putative secreted triacylglycerol lipase. Interestingly, the expression of the *H. alvei* glycerol degradation pathway via glycerol dehydrogenase considerably increased in the presence of *B. aurantiacum* (Supplementary Table S11). It may therefore be hypothesized that the glycerol liberated by the triacylglycerol lipase secreted by *B. aurantiacum* could be used by *H. alvei* as an energy source. Glycerol was detected in the mini-cheeses by HPLC, but no significant difference between the conditions investigated could be observed (Supplementary Table S3). In *B. aurantiacum* and *D. hansenii*, some differences in expression levels of genes involved in lipid catabolism were observed in the different biological conditions. However, no common pattern could be distinguished (Supplementary Tables S10, S12).

#### Iron Acquisition

**FIGURE 4:**
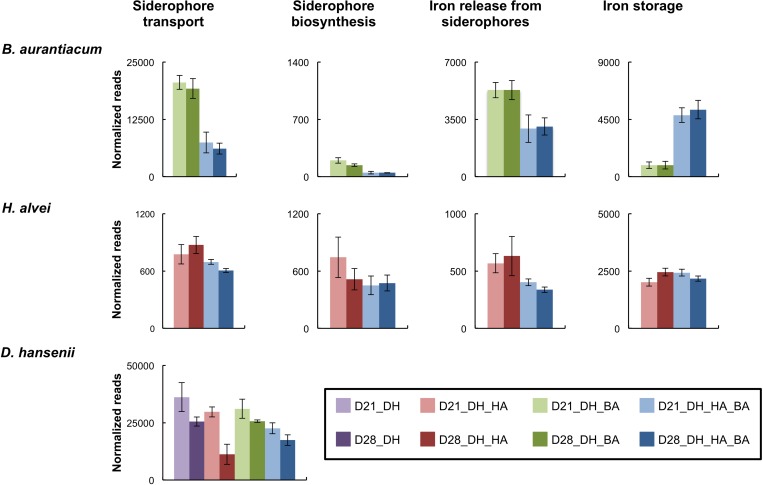
Expression of genes involved in iron acquisition in the mini-cheeses. The expression level for each type of gene is represented as the sum of the sequencing reads (normalized against each species) that mapped to the corresponding genes. Bars are colored according to the biological conditions; D21 and D28 correspond to the sampling time (day 21 and day 28, respectively); DH, HA, and BA correspond to the presence of *D. hansenii*, *H. alvei*, and *B. aurantiacum*, respectively. The error bars represent the standard deviations (four cheese replicates).

Inoculation of *H. alvei* in the mini-cheeses strongly decreased the expression of the *B. aurantiacum* siderophore biosynthesis genes, of the siderophore transporters, and of the proteins involved in the release of iron from siderophores (Figure 4 and Supplementary Table S13). This suggests that there is a higher iron availability for *B. aurantiacum* when it is co-cultivated with *H. alvei*. In addition, co-cultivation with *H. alvei* increased the expression of the *B. aurantiacum* ferritin (EC 1.16.3.2), a bacterial non-heme iron storage protein, which suggests that *H. alvei* increased the amount of intracellular iron in *B. aurantiacum*. The *H. alvei* genome encodes a hydroxamate-type siderophore biosynthesis cluster. At day 21, the expression of this cluster slightly decreased in the presence of *B. aurantiacum*, but no difference was observed for day 28 (Figure 4 and Supplementary Table S14). The presence of *B. aurantiacum* decreased the expression level of the genes encoding the periplasmic protein TonB (Ga0116594_112230) and the TonB-dependent outer membrane siderophore receptor (Ga0116594_11037). TonB belongs to a protein complex that delivers energy for the transport of Fe^3+^/siderophore complexes through the outer membrane of Gram-negative bacteria (Andrews et al., 2003). There was also a lower expression of part of the ABC-type Fe^3+^/siderophore component genes, but many of these genes had a low expression level, which explains why only a slight decrease in the global expression level (cumulated reads) was observed. The iron storage protein identified in the genome of *H. alvei* is bacterioferritin (EC 1.16.3.1), which is a heme-containing protein. Expression of the bacterioferritin gene in *H. alvei* was not modified when it was co-cultivated with *B. aurantiacum*.

In *D. hansenii*, some differences in expression levels of genes involved in iron acquisition were observed in the eight biological conditions, but no common pattern could be distinguished (Supplementary Table S15). However, at day 28, there was a large decrease of the global expression level of these genes (cumulated reads) in the mini-cheeses inoculated with *D. hansenii* and *H. alvei* in comparison to the mini-cheeses inoculated with only *D. hansenii* (Figure 4).

#### Sulfur Metabolism

**FIGURE 5:**
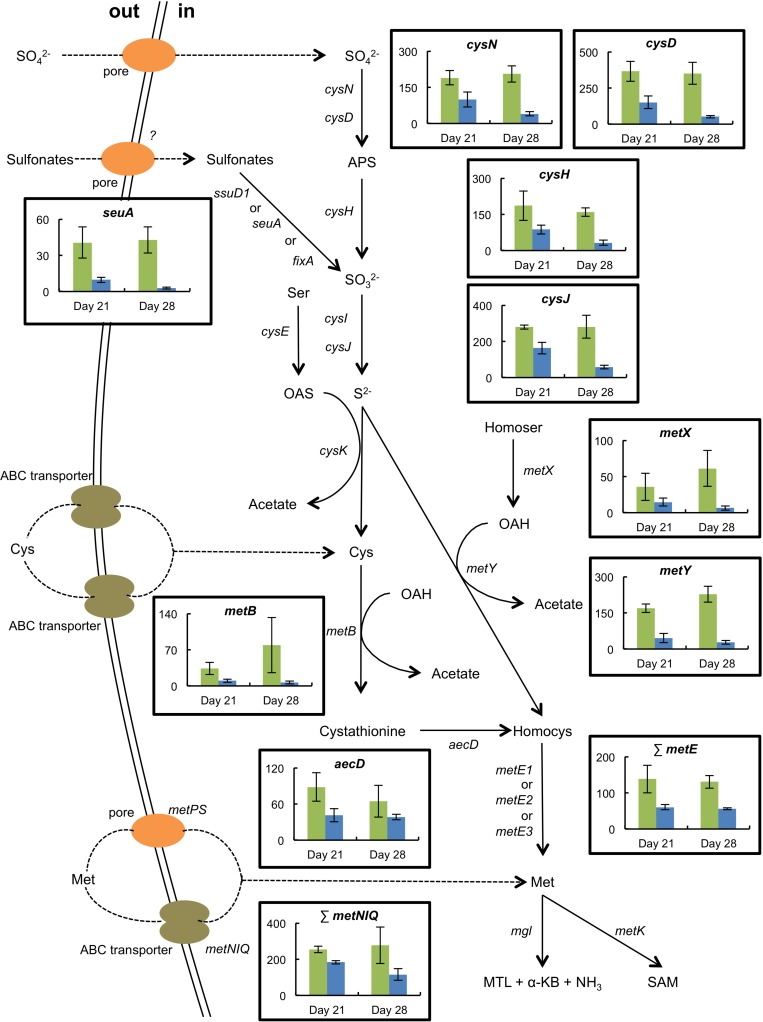
Expression of selected genes involved in sulfur metabolism in *B. aurantiacum* grown in the absence (green) or the presence (blue) of *H. alvei* (sequencing reads normalized against *B. aurantiacum*). The expression level of the methionine synthases (*metE*) or of the high-affinity methionine ABC transporter (*metNIQ*) is represented as the sum of the sequencing reads that mapped to the corresponding genes. The error bars represent the standard deviations (four cheese replicates). Transporter genes: *metPS*, low-affinity methionine transporter; *metNIQ*, high-affinity methionine ABC transporter. Metabolism genes: *cysND*, sulfate adenylyltransferase; *cysH*, adenylyl sulfate reductase; *ssuD1*, alkanesulfonate monooxygenase; *seuA*, reduced flavin mononucleotide (FMNH_2_)-dependent alkanesulfonate monooxygenase; *fixA*, electron transfer flavoprotein; *cysIJ*, sulfite reductase; *cysE*, serine *O*-acetyltransferase; *cysK*, *O*-acetylserine-thiol-lyase; *metB*, cystathionine gamma-synthase; *aecD*, cystathionine beta-lyase; *metX*, homoserine *O*-acetyltransferase; *metY*, *O*-acetylhomoserine-thiol-lyase; *metE1-3*, cobalamin-independent methionine synthase; *mgl*, methionine gamma-lyase; *metK*, *S*-adenosylmethionine synthase. Compounds: APS, adenylyl sulfate; Ser, serine; OAS, *O-*acetylserine; Cys, cysteine; Homocys, homocysteine; Homoser, homoserine; OAH, *O-*acetylhomoserine; Met, methionine; MTL, methanethiol; α-KB, alpha-ketobutyrate; SAM, *S-*adenosylmethionine.

**FIGURE 6:**
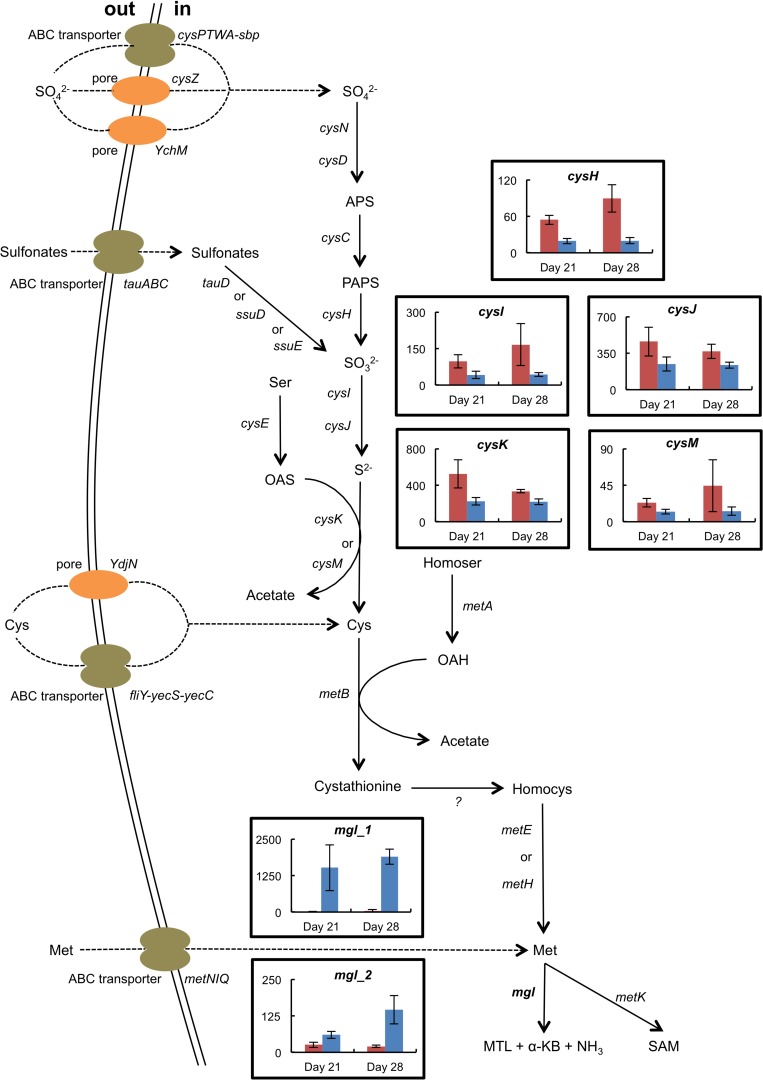
Expression of selected genes involved in sulfur metabolism in *H. alvei* grown in the absence (red) or the presence (blue) of *B. aurantiacum* (sequencing reads normalized against *H. alvei*). The error bars represent the standard deviations (four cheese replicates). *mgl_1* corresponds to Ga0116594_105962; *mgl_2* corresponds to Ga0116594_100764. Transporter genes: *cysPTWA-sbp*, sulfate ABC transporter; *YchM*, sulfate permease; *cysZ*, pH dependent sulfate transporter; *tauABC*, taurine ABC transporter; *ydjN*, cystine transporter; fliY-yecS-yecC, cystine ABC transporter; *metNIQ*, high-affinity methionine ABC transporter. Metabolism genes: *cysND*, sulfate adenylyltransferase; *cysC*, adenylylsulfate kinase; *cysH*, phosphoadenylyl-sulfate reductase; *tauD*, taurine dioxygenase; *ssuD*, alkanesulfonate monooxygenase; *ssuE*, flavin mononucleotide (FMN) reductase; *cysIJ*, sulfite reductase; *cysE*, serine *O*-acetyltransferase; *cysK*, cysteine synthase A; *cysM*, cysteine synthase B; *metA*, homoserine *O*-succinyltransferase; *metB*, cystathionine gamma-synthase; *metE*, cobalamin-independent methionine synthase; *metH*, cobalamin-dependent methionine synthase; *mgl*, methionine gamma-lyase; *metK*, *S*-adenosylmethionine synthase. Compounds: APS, adenylyl sulfate; PAPS, 3′-Phosphoadenosine-5′-phosphosulfate; Ser, serine; OAS, *O-*acetylserine; Cys, cysteine; Homocys, homocysteine; Homoser, homoserine; OAH, *O-*acetylhomoserine; Met, methionine; MTL, methanethiol; α-KB, alpha-ketobutyrate; SAM, *S-*adenosylmethionine.

The presence of *H. alvei* in the mini-cheeses decreased the expression of many *B. aurantiacum* genes involved in sulfate and sulfonate assimilation (*cysN*, *cysD*, *cysH*, *cysJ*, *cysG*, and *seuA*), in methionine biosynthesis (*metX*, *metY*, *metB*, *aecD*, and *metE2*), and in methionine import (*metNIQ*), especially at day 28 (Supplementary Table S16 and Figure 5). This suggests that there was a lower demand for sulfur amino acids in *B. aurantiacum* when it was co-cultivated with *H. alvei*. A previous study revealed that a cluster encoding an oligopeptide ABC transporter in *B. aurantiacum* ATCC 9175^*T*^ is up-regulated during sulfur depletion (Forquin et al., 2011). This cluster corresponds to the locus tags Ga0063698_03331 to 03334 of the *B. aurantiacum* strain used in the present study. Interestingly, at day 28, the cluster was down-regulated when *B. aurantiacum* was co-cultivated with *H. alvei*, suggesting that more sulfur was available for *B. aurantiacum* in that condition. Concerning the expression of *H. alvei* genes involved in sulfur metabolism, we observed that several genes involved in sulfate assimilation (*cysH*, *cysI*, and *cysJ*) and in cysteine biosynthesis (*cysK*, *cysM*) were down-regulated in the presence *B. aurantiacum*, and, as already mentioned, *B. aurantiacum* considerably increased *H. alvei mgl* expression (Supplementary Table S17 and Figure 6). This suggests that *B. aurantiacum* increased the availability of sulfur amino acids for *H. alvei.* In *D. hansenii*, some differences in the expression levels of genes involved in sulfur metabolism were observed across the eight biological conditions, but no common pattern could be distinguished (Supplementary Table S18).

## Discussion

Ripening cultures are widely used in the production of smear-ripened cheeses. Understanding the biotic interactions within these cultures could help to improve strategies for the selection of strains and their associations. In this study, we investigated the biotic interactions of a quite simple cheese-ripening community composed of *D. hansenii*, *B. aurantiacum* and *H. alvei*, in a lab-scale mini-cheese model that mimics cheese-ripening conditions. Mini-cheeses were produced using all possible combinations of the three investigated microorganisms, except those in which *D. hansenii* was omitted since no growth of the bacteria occurred in that case due to the acid-sensitivity of the bacteria. In smear-ripened cheeses, the positive impact of the pH increase initiated by fungi on the growth of the more acid-sensitive bacteria is well documented (Corsetti et al., 2001). Quantification of *B. aurantiacum* and *H. alvei* in the cheese samples showed that there was a mutualistic interaction between these two bacteria. Interestingly, *H. alvei* stimulated *B. aurantiacum* despite the fact that its growth level was much lower than that of *B. aurantiacum* (1.3 × 10^8^ CFU/g at day 28 when co-cultured with *B. aurantiacum*, which represented less than 1% of the *B. aurantiacum* cell count).

We used dual RNA-seq, which makes it possible to simultaneously capture the transcriptomes of two or more interacting partners (Wolf et al., 2018), to study the interactions in the investigated microbial communities. The transcriptomic analyses revealed several potential metabolic interactions, which are summarized in Table 4. Cheese is a highly iron-restricted habitat and it was also demonstrated that iron availability can be a limiting factor in the growth of typical ripening bacteria at the cheese surface (Monnet et al., 2012). In *Brevibacterium* strains, several horizontal gene transfer clusters involved in iron acquisition have been described (Bonham et al., 2017; Pham et al., 2017), and recently, a plasmid encoding putative iron import proteins was described in a strain isolated from an Austrian hard cheese rind (Anast et al., 2019). Co-cultivation of *B. aurantiacum* with *H. alvei* strongly increased the expression of its ferritin gene and strongly decreased the expression of most of its genes involved in iron acquisition, which includes siderophore biosynthesis genes and genes coding for ABC-type Fe^3+^/siderophore components and for siderophore interacting proteins. The main function of ferritin is to store iron in the cells, and it was shown that in *Corynebacterium glutamicum*, this gene is induced under high-iron conditions (Brune et al., 2006; Wennerhold and Bott, 2006). In addition, siderophore biosynthesis, siderophore transport and siderophore interacting proteins are generally repressed in high-iron conditions (Wandersman and Delepelaire, 2004). This suggests that *H. alvei* provided iron to *B. aurantiacum*, probably by means of its siderophore production. Indeed, the *H. alvei* genome contains a gene cluster encoding a hydroxamate-type siderophore. Appropriation of siderophores excreted by other organisms is a strategy that has been described in various microorganisms. For the recipient bacterium, such siderophores are known as “xenosiderophores” (Sheldon and Heinrichs, 2015). The capacity of cheese-associated *Brevibacterium* strains to use xenosiderophores produced by other *Brevibacterium* strains was demonstrated *in vitro* (Noordman et al., 2006). In addition, stimulation of *Staphylococcus equorum* by the fungus *Scopulariopsis* was observed in experimental communities composed of cheese-associated strains, and was attributed to fungal siderophore production that reduces the high production cost of the siderophore staphyloferrin B by *S. equorum* (Kastman et al., 2016). There is thus increasing evidence that iron acquisition plays an important role in the biotic interactions between cheese surface microorganisms.

**TABLE 4:** Possible biotic interactions between *D. hansenii*, *H. alvei*, and *B. aurantiacum* in the mini-cheeses.

**Metabolism**	**Interaction between species**
Deacidification^a^	• Deacidification of cheese by *D. hansenii* favors the growth of *B. aurantiacum* and *H. alvei*, which are more acid-sensitive than the yeast.
Iron metabolism^b^	• Siderophore production by *H. alvei* increases iron availability for *B. aurantiacum.*
Lipolysis^b,c,d^	• Lipolytic activity of *B. aurantiacum* on triglycerides increases the release of glycerol in cheese that can be used by *H. alvei* as an energy substrate. The fatty acids released from triglycerides may also be used by *H. alvei*, but no differences were observed in the expression of beta-oxidation genes.
Proteolysis^b,c,d^	• Proteolytic activity of *B. aurantiacum* on caseins increases the nitrogen availability in cheese for *D. hansenii* and *H. alvei.*
Sulfur metabolism^b,d^	• The presence of *H. alvei* increases the sulfur amino acid availability for *B. aurantiacum*.
D-galactonate^b,d^	• Possible production of D-galactonate by oxidation of lactose/galactose and subsequent use as an energy source by *B. aurantiacum* and *H. alvei.*

*^*a*^Interaction deduced from growth in the mini-cheeses. ^*b*^Interaction deduced from transcriptomic data. ^*c*^Interaction deduced from proteolytic/lipolytic assays on agar plates. ^*d*^Interaction deduced from UHPLC-MS or HPLC-UV analyses.*

Our results suggest that *H. alvei* could benefit from the lipolytic activity of *B. aurantiacum*. This was deduced from lipolytic plate assay, genomic analysis and transcriptomic analysis of the mini-cheeses. Indeed, no lipolytic activity was detected for *H. alvei* on tributyrin agar, and no gene encoding putative secreted triacylglycerol lipase was identified in its genome. In the presence of *B. aurantiacum*, there was a strong up-regulation of *H. alvei* genes involved in the catabolism of glycerol, an energy substrate derived from triglyceride lipolysis. We may thus hypothesize that the growth of *H. alvei* is favored by the glycerol released from the cheese triglycerides through the action of the triacylglycerol lipase secreted by *B. aurantiacum.*

Another potential mechanism of biotic interactions in the mini-cheeses is the proteolytic activity of *B. aurantiacum.* Indeed, the investigated *B. aurantiacum* strain showed proteolytic activity on calcium caseinate agar, whereas no activity was detected for *D. hansenii* and *H. alvei*. In addition, transcriptomic data suggested that the presence of *B. aurantiacum* increased nitrogen availability in the mini-cheeses for *H. alvei* and *D. hansenii*. This was confirmed by the measurement of free amino acid concentration. One major change of the *H. alvei* transcriptome when it was co-cultured with *B. aurantiacum* was an increased expression of the methionine catabolism pathway via methionine gamma-lyase. In several bacteria such as *B. aurantiacum* (Forquin et al., 2011), *Pseudomonas putida* (Inoue et al., 1997), and *Citrobacter freundii* (Manukhov et al., 2006), it has been shown that the expression of the methionine gamma-lyase gene is induced by methionine. The methanethiol released in this reaction is a precursor for a wide variety of volatile sulfur compounds (VSCs) that contribute to cheese flavor (Bonnarme et al., 2001). Our results suggest that the catabolism of methionine by *H. alvei* and, in consequence, its production of VSCs, are stimulated by the proteolytic activity of other cheese microorganisms, which may have practical applications for a better control of the aroma compound production by *H. alvei* in cheese-making processes.

The transcriptomic data revealed that *H. alvei* strongly repressed the expression of the sulfur metabolism genes in *B. aurantiacum*, suggesting that the presence of *H. alvei* increased sulfur amino acid availability in *B. aurantiacum*. This is not easy to explain since no proteolytic activity was observed for *H. alvei*. It may be hypothesized that in the absence of *H. alvei*, the poor growth of *B. aurantiacum* that resulted from the low iron availability also limited the amount of proteases secreted in the cheese, leading to a low amount of free sulfur amino acids in the cheese. More experiments are needed to confirm or invalidate this hypothesis.

The present study also indicated that the D-galactonate catabolism genes of *B. aurantiacum* and *H. alvei* are expressed during their growth in cheese. To our knowledge, the presence of D-galactonate in cheeses had never been investigated before. However, it was hypothesized that in some cheese varieties, this compound could be produced by the oxidation of residual lactose or galactose, and subsequently used as a growth substrate by some cheese microorganisms such as *Glutamicibacter arilaitensis* (Monnet et al., 2010). In the present study, we were able to detect galactonate in our mini-cheeses, which confirms that this compound can be produced by some cheese microorganisms. D-galactonate production via a versatile L-arabinose dehydrogenase (AraDH) from *Azospirillum brasilense* was demonstrated in an engineered *E. coli* strain (Liu et al., 2014). Interestingly, the genome of the yeast *D. hansenii* encodes a putative arabinose dehydrogenase (locus tag DEHA2B12980g), which might explain galactonate production in cheese by this microorganism.

**FIGURE 7:**
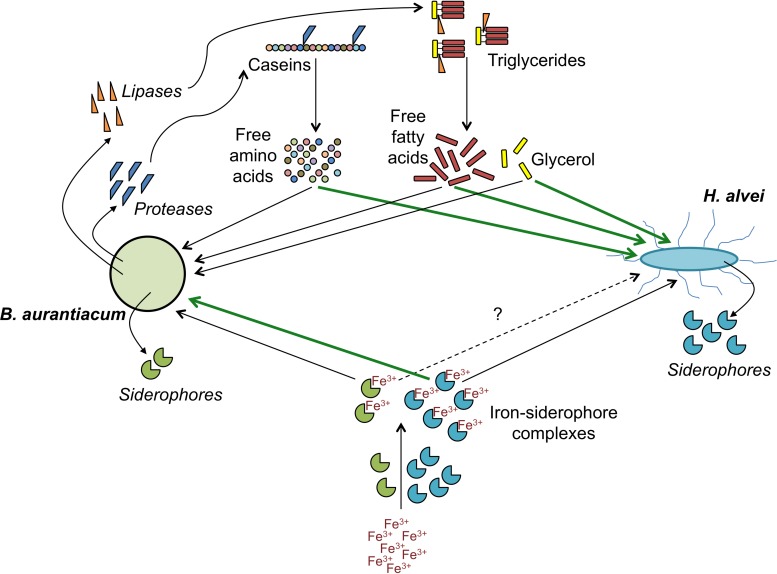
Proposed mechanisms involved in the mutualistic relationship between *B. aurantiacum* and *H. alvei.* Green lines indicate processes by which a species could stimulate its partner.

In conclusion, this study illustrated the interest of dual RNA-seq analyses to provide insight into the molecular bases of biotic interactions between cheese surface microorganisms. By combining microbial, biochemical and transcriptomic analyses of lab-scale mini-cheeses, we identified potential mechanisms underlying the mutualistic interaction between *B. aurantiacum* 8(6) and *H. alvei* GB001. When *H. alvei* is co-cultivated with *B. aurantiacum*, the latter is probably stimulated by the siderophores produced by *H. alvei* and, in parallel, it seems to stimulate *H. alvei* through the secretion of proteases and lipases, which generate energy substrates (Figure 7). Several examples of microbial communities containing strains that use siderophores released by other strains have been reported (Sheldon and Heinrichs, 2015). This behavior, often referred to as “cheating,” probably represents an adaptive trait in environments in which siderophore production is functionally redundant. However, in the present example, there is, instead, a mutualistic interaction between the siderophore producer and its recipient. In the future, it would be interesting to take the iron acquisition systems of cheese-associated strains into account for the purpose of improving the selection of the ripening culture components and their association in mixed cultures. In addition, it is possible that the iron metabolism of ripening cultures can influence the growth of adventitious strains or of pathogens at the cheese surface.

## Data Availability

The datasets generated for this study can be found in the raw Illumina data for all the samples were deposited in the European Nucleotide Archive of the European Bioinformatics Institute under the accession number PRJEB30420.

## Author Contributions

N-PP and CM wrote the manuscript. N-PP, SL, PB, and CM analyzed the transcriptomic data. CM coordinated the study. PL performed the metabolomic analyses. All authors read and approved the final manuscript.

## Conflict of Interest Statement

The authors declare that the research was conducted in the absence of any commercial or financial relationships that could be construed as a potential conflict of interest.

## Footnotes

1 http://migale.jouy.inra.fr/galaxy/
