# Supplementary material for: Transcription Profiling Reveals Cooperative Metabolic Interactions in a Microbial Cheese-Ripening Community Composed of Debaryomyces hansenii, Brevibacterium aurantiacum, and Hafnia alvei
Source: Front Microbiol. 2019 Aug 16;10:1901. doi: 10.3389/fmicb.2019.01901 (PMC6706770; doi:10.3389/fmicb.2019.01901)
Supplement: Supplementary file 1 [file Table_1.DOCX]

**TABLE S1 |** Number and proportion of CDSs from the reference genomes that were detected.

| **Species** | **Biological condition^a^** | **CDSs detected^b^** | |
| --- | --- | --- | --- |
|  |  | **Nb** | **%** |
| *D. hansenii* | D21_DH | 6,051 | 96.28 |
|  | D28_DH | 6,054 | 96.32 |
|  | D21_DH_HA | 6,044 | 96.17 |
|  | D28_DH_HA | 6,044 | 96.17 |
|  | D21_DH_BA | 6,063 | 96.47 |
|  | D28_DH_BA | 6,047 | 96.21 |
|  | D21_DH_HA_BA | 6,050 | 96.26 |
|  | D28_DH_HA_BA | 6,020 | 95.78 |
| *H. alvei* | D21_DH_HA | 3,895 | 85.93 |
|  | D28_DH_HA | 3,955 | 87.25 |
|  | D21_DH_HA_BA | 3,752 | 82.77 |
|  | D28_DH_HA_BA | 3,952 | 87.18 |
| *B. aurantiacum* | D21_DH_BA | 2,979 | 72.82 |
|  | D28_DH_BA | 2,664 | 65.12 |
|  | D21_DH_HA_BA | 3,523 | 86.12 |
|  | D28_DH_HA_BA | 3,524 | 86.14 |

^a^Biological condition: D21 and D28 correspond to the sampling time (day 21 and day 28, respectively); DH, HA and BA correspond to the presence of *D. hansenii*, *H. alvei* and *B. aurantiacum*, respectively.

^b^≥ten reads per CDS across all replicates.

**TABLE S2 |** Effect of *H. alvei* and of *B. aurantiacum* on the transcriptome of the other microorganisms^a^.

|  | ***B. aurantiacum*** | | ***H. alvei*** | | ***D. hansenii*** | |
| --- | --- | --- | --- | --- | --- | --- |
|  | **Day 21** | **Day 28** | **Day 21** | **Day 28** | **Day 21** | **Day 28** |
| **Effect of *H. alvei*** | | | | | | |
|  | D21_DH_HA_BA  *vs.* D21_DH_BA | D28_DH_HA_BA  *vs.* D28_DH_BA |  |  | D21_DH_HA  *vs.* D21_DH | D28_DH_HA  *vs.* D28_DH |
| Genes up-regulated^b^ | 90 | 166 |  |  | 5 | 57 |
| Genes down-regulated^c^ | 125 | 149 |  |  | 3 | 94 |
| **Effect of *B. aurantiacum*** | | | | | | |
|  |  |  | D21_DH_HA_BA  *vs.* D21_DH_HA | D28_DH_HA_BA  *vs.* D28_DH_HA | D21_DH_BA  *vs.* D21_DH | D28_DH_BA  *vs.* D28_DH |
| Genes up-regulated^b^ |  |  | 407 | 521 | 5 | 25 |
| Genes down-regulated^c^ |  |  | 197 | 279 | 29 | 55 |
| **Effect of the combination of *H. alvei* and *B. aurantiacum*** | | | | | | |
|  |  |  |  |  | D21_DH_HA_BA  *vs.* D21_DH | D28_DH_HA_BA  *vs.* D28_DH |
| Genes up-regulated^b^ |  |  |  |  | 327 | 590 |
| Genes down-regulated^c^ |  |  |  |  | 177 | 366 |

^a^Biological conditions are coded according to the following rules: D21 and D28 correspond to the sampling time (day 21 and day 28, respectively); DH, HA and BA correspond to the presence of *D. hansenii*, *H. alvei* and *B. aurantiacum*, respectively.

^b^*p* < 0.05 according to DESeq2 and fold change ≥ 2.

^c^*p* < 0.05 according to DESeq2 and fold change ≤ 0.5.

**TABLE S3 |** Quantification of metabolites present in the mini-cheeses after 28 days of ripening*.

| **Metabolites**** | **Concentration***** | | | | | |
| --- | --- | --- | --- | --- | --- | --- |
|  | **Initial curd** | **DH** | **DH_HA** | **DH_BA** | **DH_HA_BA** | |
| ***Quantification by UHPLC-MS (concentration in µmol.kg^-1^)*** | | | | | |  |
| *Amino acids* | | | | | |  |
| Aspartic acid | 175.0 ± 27.7^a^ | 12.8 ± 2.7^d^ | 18.4 ± 3.9^d^ | 25.2 ± 1.2^cd^ | 218.4 ± 49.0^a^ | |
| Serine | 36.7 ± 5.6^b^ | 12.1 ± 1.8^b^ | 13.5 ± 2.2^b^ | 28.4 ± 1.8^b^ | 73.9 ± 17.4^a^ | |
| Glycine | 37.8 ± 6.1^b^ | 11.7 ± 0.8^c^ | 15.5 ± 1.2^c^ | 17.8 ± 1.5^c^ | 55.7 ± 9.5^a^ | |
| Cysteine | ND | ND | ND | ND | ND | |
| Threonine | 52.6 ± 9.4^bc^ | 15.7 ± 2.6^c^ | 16.8 ± 1.5^c^ | 34.4 ± 1.5^bc^ | 112.8 ± 15.9^a^ | |
| Glutamic acid | 238.6 ± 41.3^c^ | 36.5 ± 9.1^c^ | 128.5 ± 39.7^c^ | 133.0 ± 16.6^c^ | 1500.7 ± 111.5^a^ | |
| Alanine | 232.7 ± 37.4^c^ | 69.6 ± 1.9^c^ | 75.2 ± 12.5^c^ | 144.0 ± 7.2^c^ | 917.8 ± 88.0^a^ | |
| Proline | 670.0 ± 118.5^a^ | 27.8 ± 0.8^d^ | 38.3 ± 5.0^d^ | 75.9 ± 6.4^cd^ | 200.8 ± 26.0^bc^ | |
| Valine | 233.0 ± 45.7^bc^ | 72.3 ± 17.1^c^ | 54.3 ± 21.8^c^ | 410.6 ± 21.1^b^ | 1497.0 ± 243.3^a^ | |
| Histidine | 314.1 ± 41.4^a^ | 27.1 ± 3.6^bc^ | 15.9 ± 2.6^c^ | 41.0 ± 4.8^bc^ | 74.5 ± 18.5^b^ | |
| Methionine | 14.7 ± 2.9^c^ | 9.9 ± 2.4^c^ | 10.7 ± 4.0^c^ | 31.1 ± 6.2^bc^ | 79.0 ± 13.2^a^ | |
| Lysine | 701.4 ± 134.1^a^ | 29.6 ± 9.3^c^ | 5.5 ± 1.8^c^ | 65.2 ± 7.1^bc^ | 35.3 ± 24.5^c^ | |
| Arginine | 2.8 ± 0.6^c^ | 2.8 ± 0.6^c^ | 1.5 ± 0.6^c^ | 6.3 ± 1.3^bc^ | 14.4 ± 6.0^b^ | |
| Tyrosine | 301.2 ± 46.0^a^ | 19.4 ± 3.0^c^ | 12.0 ± 3.4^c^ | 56.7 ± 2.3^c^ | 139.0 ± 35.5^b^ | |
| Isoleucine | 79.8 ± 14.7^bc^ | 31.7 ± 7.7^c^ | 23.3 ± 9.7^c^ | 141.5 ± 4.9^b^ | 388.3 ± 64.8^a^ | |
| Leucine | 419.5 ± 82.5^b^ | 50.8 ± 8.9^b^ | 37.1 ± 6.8^b^ | 698.8 ± 35.3^b^ | 3266.9 ± 688.8^a^ | |
| Phenylalanine | 215.3 ± 32.1^b^ | 26.9 ± 4.4^d^ | 18.7 ± 2.7^d^ | 150.4 ± 8.6^bc^ | 316.6 ± 74.8^a^ | |
| Asparagine | 201.4 ± 33.8^a^ | 16.7 ± 2.5^c^ | 0.1 ± 0.1^c^ | 30.7 ± 1.5^bc^ | 0.3 ± 0.0^c^ | |
| Glutamine | 35.7 ± 4.4^cd^ | 36.9 ± 4.8^cd^ | 8.2 ± 2.5^d^ | 59.3 ± 3.5^bc^ | 30.6 ± 4.2^cd^ | |
| *Sulfur metabolites* | | | | | |  |
| Taurine | ND | ND | ND | ND | ND | |
| Hypotaurine | ND | ND | ND | ND | ND | |
| Homocysteine | ND | ND | ND | ND | ND | |
| OAS | 7.7 ± 1.3 | ND | ND | ND | ND | |
| Cysteine sulfinic acid | ND | ND | ND | ND | ND | |
| Glutathione | ND | ND | ND | ND | ND | |
| SAM | ND | ND | ND | ND | ND | |
| Cys-Gly | ND | ND | ND | ND | ND | |
| Cystathionine | 12.8 ± 2.1^bc^ | 17.8 ± 3.6^ab^ | 12.1 ± 3.1^bc^ | 22.4 ± 5.1^a^ | 10.3 ± 1.4^bc^ | |
| γ-Glu-Cys | ND | ND | ND | ND | ND | |
| SAH | ND | ND | ND | ND | ND | |
| *Polyamines* | | | | | |  |
| Histamine | ND | ND | ND | ND | ND | |
| Tyramine | ND | ND | ND | ND | ND | |
| Phenylethylamine | ND | ND | ND | ND | ND | |
| Putrescine | ND | ND | ND | ND | ND | |
| Cadaverine | ND | ND | 86.8 ± 30.6^b^ | ND | 211.7 ± 40.7^a^ | |
| Spermine | ND | ND | ND | ND | ND | |
| Spermidine | ND | ND | ND | ND | ND | |
| *Others* | | | | | |  |
| Galactonate | 10.3 ± 0.3^d^ | 56.0 ± 3.1^a^ | 21.7 ± 7.9^cd^ | 26.0 ± 3.7^bc^ | 60.8 ± 6.3^a^ | |
| Ornithine | 163.8 ± 27.4^a^ | 2.8 ± 0.3^b^ | 1.0 ± 0.5^b^ | 3.7 ± 0.3^b^ | 5.5 ± 1.4^b^ | |
| ***Quantification by HPLC (concentration in mmol.kg^-1^)*** | | | | | |  |
| Lactose | 6.6 ± 1.4 | ND | ND | ND | ND | |
| Galactose | 1.4 ± 0.4 | ND | ND | ND | ND | |
| Lactic acid | 105.4 ± 21.1 | ND | ND | ND | ND | |
| Citric acid | 8.3 ± 0.9^a^ | 4.5 ± 0.4^b^ | 0.8 ± 0.2^c^ | 4.2 ± 0.5^b^ | 0.5 ± 0.1^c^ | |
| Acetic acid | 2.2 ± 0.3^a^ | ND | ND | 0.9 ± 0.8^ab^ | 2.4 ± 0.4^a^ | |
| Ethanol | ND | ND | ND | ND | ND | |
| Glycerol | 2.5 ± 2.2^a^ | 2.8 ± 0.8^a^ | 7.0 ± 3.2^a^ | 10.2 ± 5.3^a^ | 5.1 ± 1.9^a^ | |

^*^Biological conditions are coded according to the following rules: DH, HA and BA correspond to the presence of *D. hansenii*, *H. alvei* and *B. aurantiacum*, respectively.

^**^Abbreviations: OAS, *O-*acetylserine; SAM, *S-*adenosylmethionine; Cys-Gly, Cysteinylglycine; γ-Glu-Cys, Gamma-glutamylcysteine; SAH, *S-*adenosylhomocysteine.

^***^Values are the arithmetic mean (*N* = 4) ± standard deviation. Mean values in the same row not followed by the same letter are significantly different at *P* < 0.05 (analysis of variance (ANOVA)). ND: not detected.
