# Supplementary material for: Transcription Profiling Reveals Cooperative Metabolic Interactions in a Microbial Cheese-Ripening Community Composed of Debaryomyces hansenii, Brevibacterium aurantiacum, and Hafnia alvei
Source: Front Microbiol. 2019 Aug 16;10:1901. doi: 10.3389/fmicb.2019.01901 (PMC6706770; doi:10.3389/fmicb.2019.01901)
Supplement: Supplementary file 3 [file Data_Sheet_1.PDF]

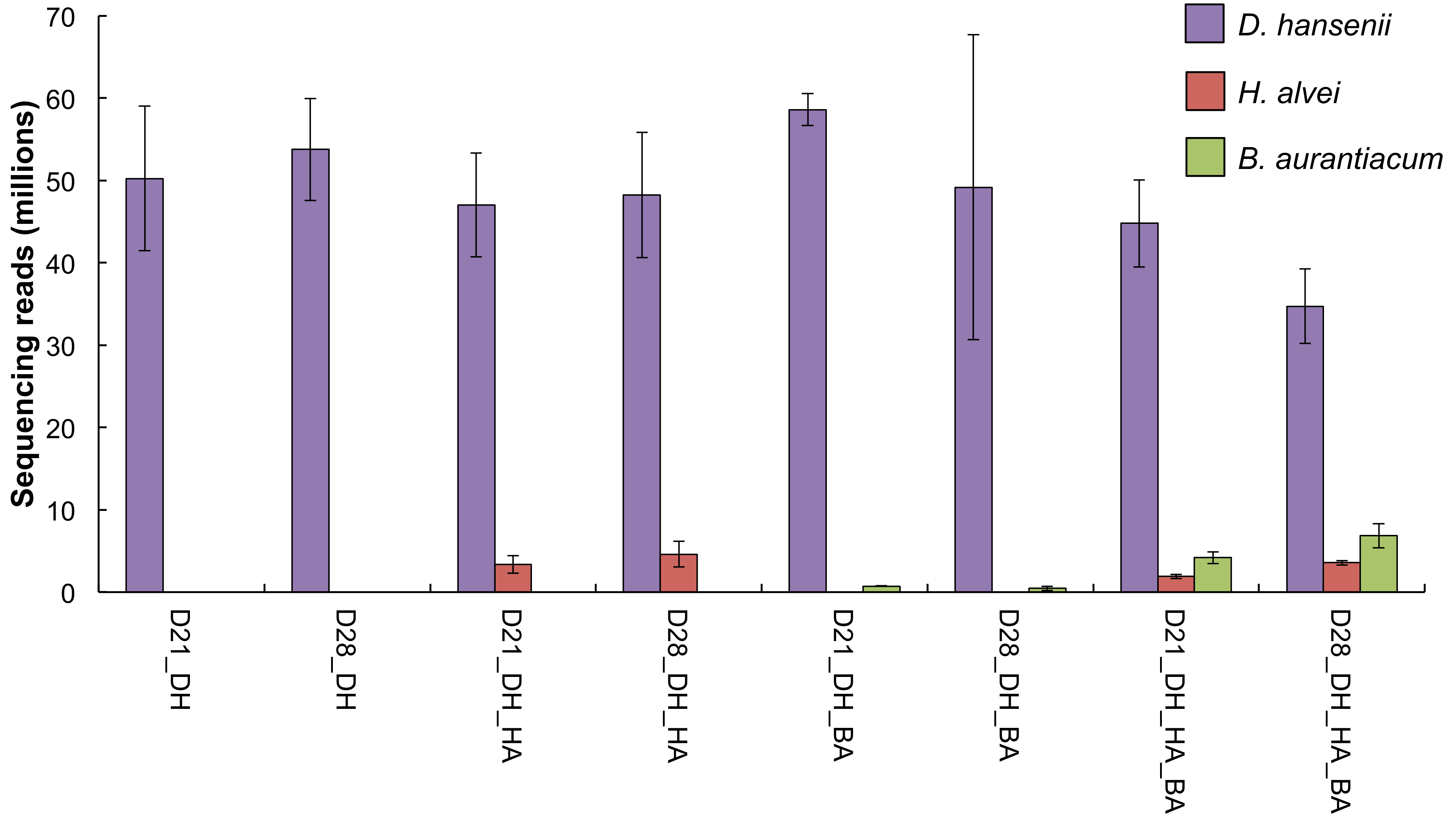

FIGURE S1 | Distribution of the sequencing reads that mapped to unique sequences of the CDSs databases established from the reference genomes. The error bars represent the standard deviations (four cheese replicates). Biological conditions are coded according to the following rules: D21 and D28 correspond to the sampling time (day 21 and day 28, respectively); DH, HA and BA correspond to the presence of *D. hansenii*, *H. alvei* and *B. aurantiacum*, respectively.

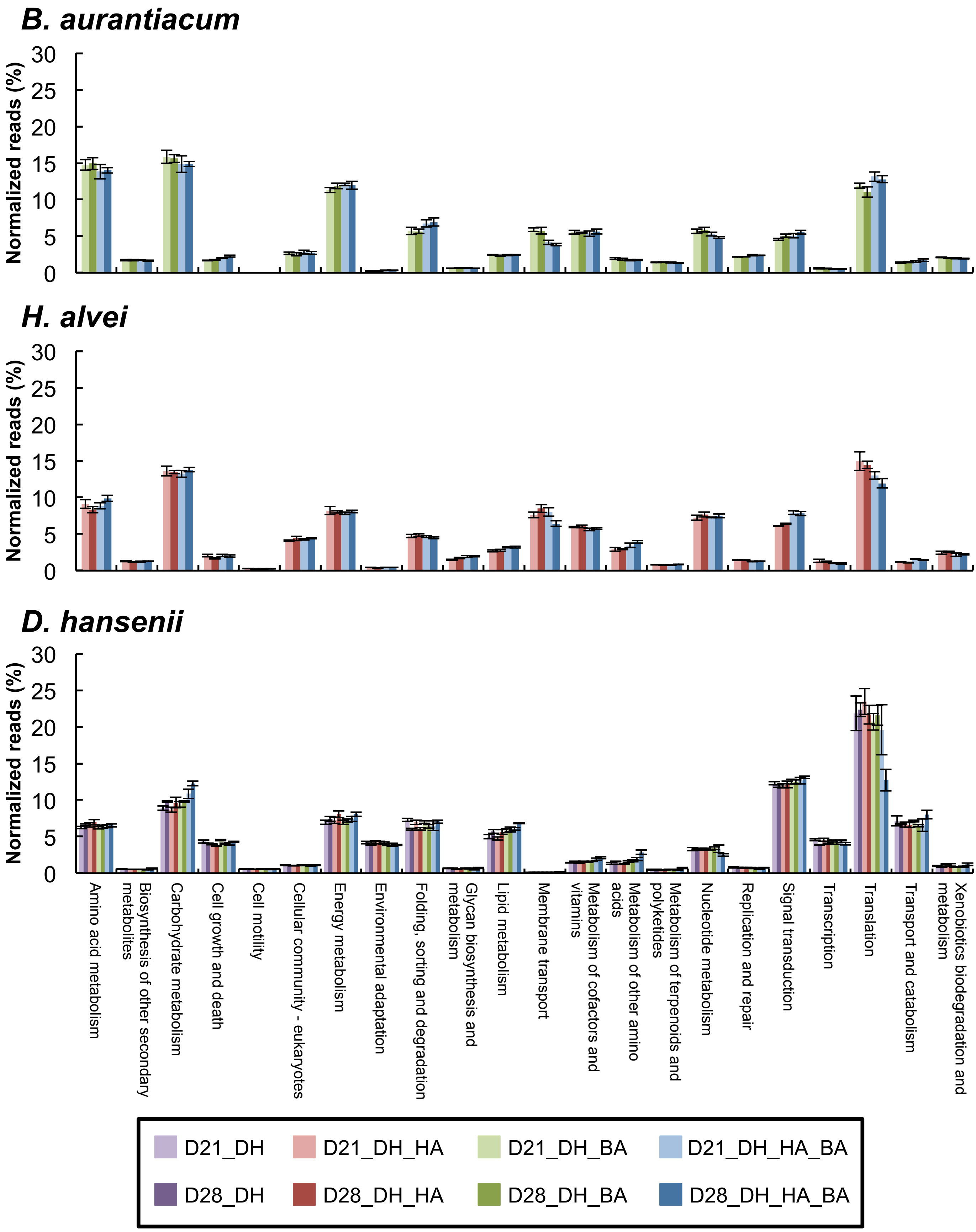

FIGURE S2 | Functional classification of the transcriptomes of *B. aurantiacum*, *H. alvei* and *D. hansenii* in the mini-cheeses. The functional classes were determined according to KEGG annotations of the CDSs. The number of reads was represented as a percentage of the total reads of the strains with KEGG assignments at each biological condition. Bars are colored according to the biological conditions; D21 and D28 correspond to the sampling time (day 21 and day 28, respectively); DH, HA and BA correspond to the presence of *D. hansenii*, *H. alvei* and *B. aurantiacum*, respectively. The error bars represent the standard deviations (four cheese replicates).

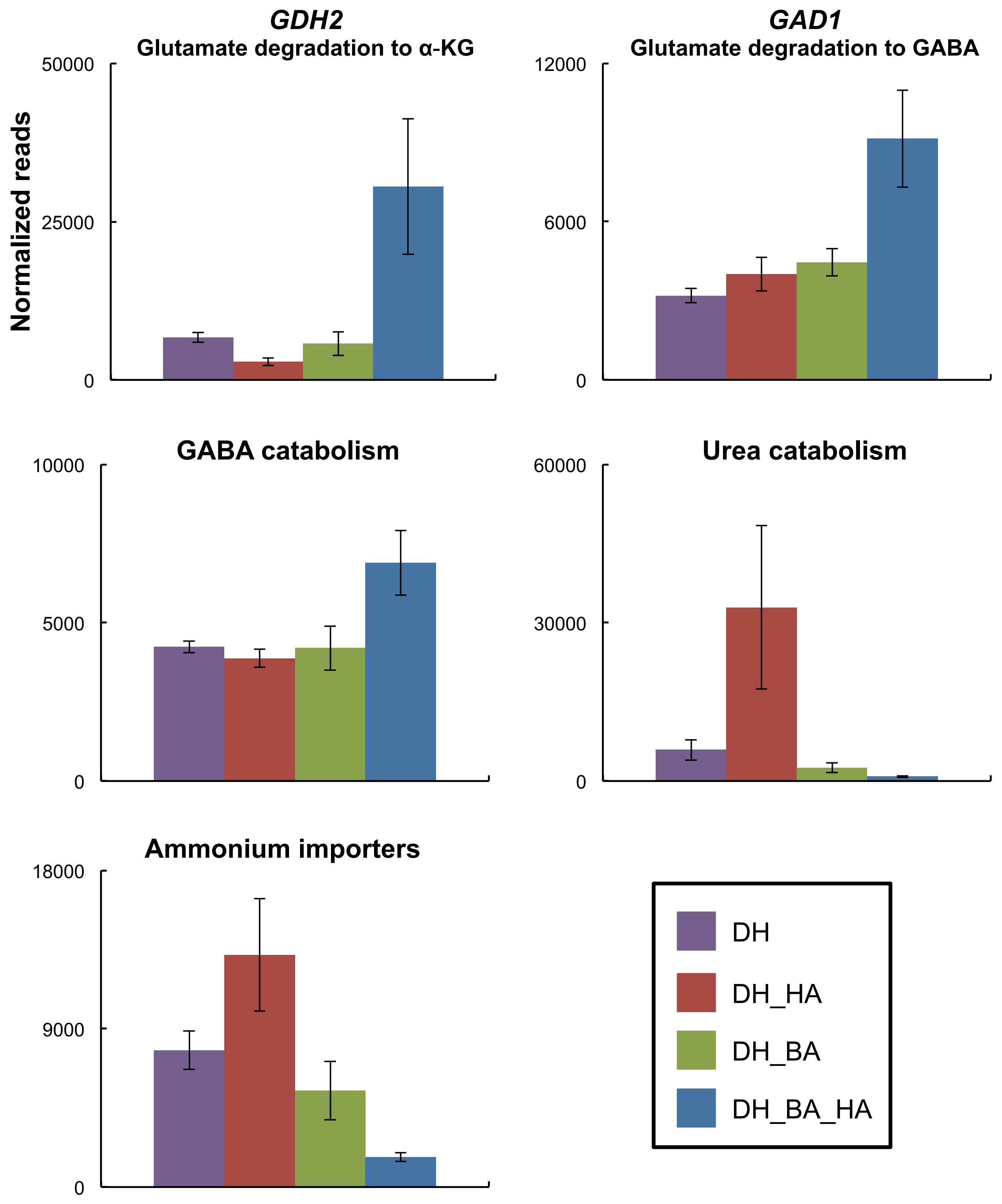

FIGURE S3 | Expression of selected genes involved in nitrogen catabolism in *D. hansenii* at day 28. The expression level of the GABA catabolism or urea catabolism pathway is represented as the sum of the sequencing reads (normalized against *D. hansenii*) that mapped to the genes of the corresponding pathway. The expression level of the ammonium importers is represented as the sum of the sequencing reads that mapped to the corresponding genes. Bars are colored according to the biological conditions; DH, HA and BA correspond to the presence of *D. hansenii*, *H. alvei* and *B. aurantiacum,* respectively. The error bars represent the standard deviations (four cheese replicates). α-KG, alpha-ketoglutarate; GABA, gamma-aminobutyrate.

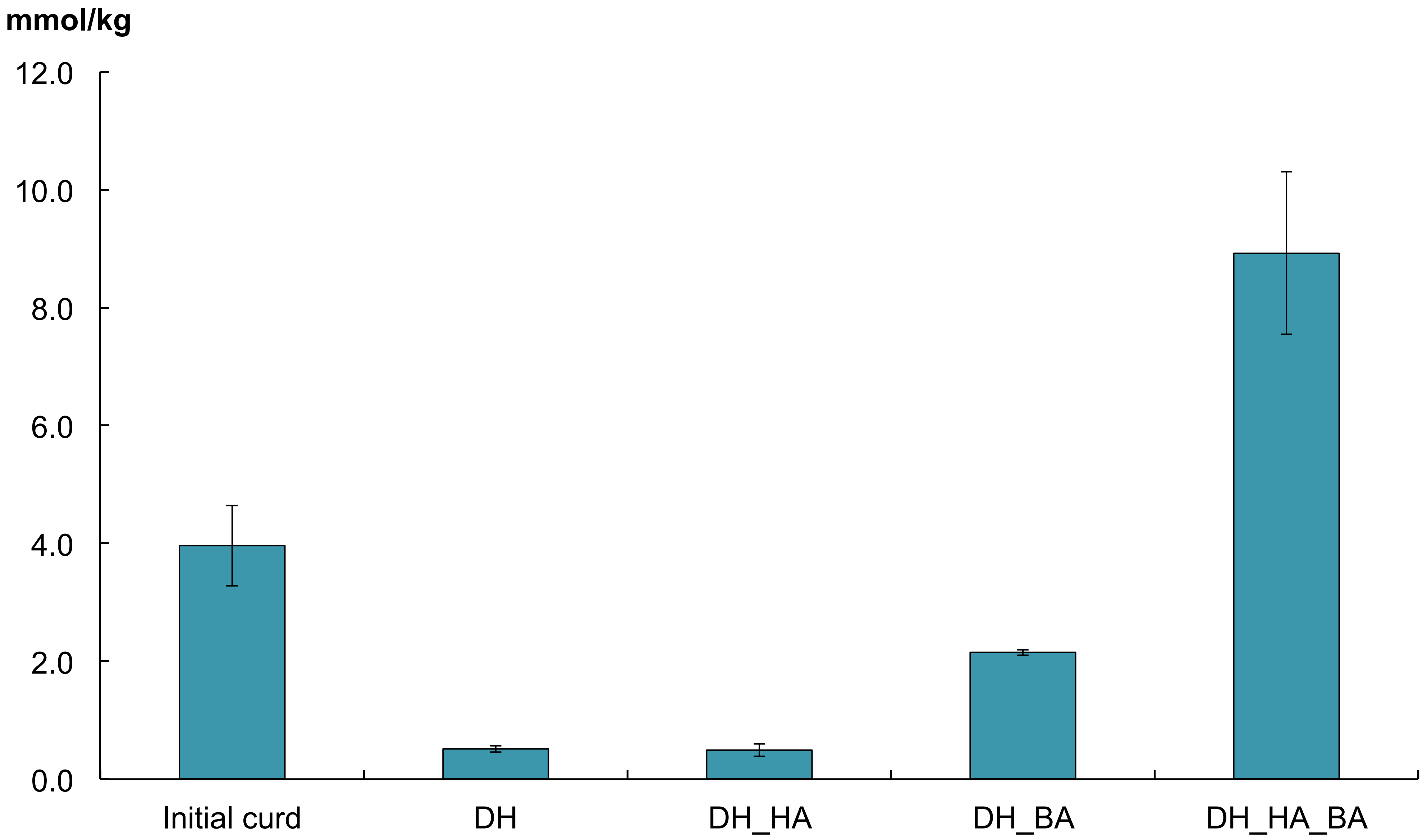

FIGURE S4 | Total concentration of free amino acids in the mini-cheeses after 28 days of ripening. The error bars represent the standard deviations (four cheese replicates). Biological conditions are coded according to the following rules: DH, HA and BA correspond to the presence of *D. hansenii*, *H. alvei* and *B. aurantiacum*, respectively.
